# Mangiferin protects against adverse skeletal muscle changes and enhances muscle oxidative capacity in obese rats

**DOI:** 10.1371/journal.pone.0173028

**Published:** 2017-03-02

**Authors:** Luz M. Acevedo, Ana I. Raya, Julio M. Martínez-Moreno, Escolástico Aguilera–Tejero, José-Luis L. Rivero

**Affiliations:** 1 Laboratory of Muscular Biopathology, Department of Comparative Anatomy and Pathological Anatomy, University of Cordoba, Cordoba, Spain; 2 Department of Animal Medicine and Surgery, University of Cordoba, Cordoba, Spain; 3 Instituto Maimonides de Investigacion Biomedica de Cordoba (IMIBIC), Hospital Universitario Reina Sofia, University of Cordoba, Cordoba, Spain; University of Birmingham, UNITED KINGDOM

## Abstract

Obesity-related skeletal muscle changes include muscle atrophy, slow-to-fast fiber-type transformation, and impaired mitochondrial oxidative capacity. These changes relate with increased risk of insulin resistance. Mangiferin, the major component of the plant *Mangifera indica*, is a well-known anti-inflammatory, anti-diabetic, and antihyperlipidemic agent. This study tested the hypothesis that mangiferin treatment counteracts obesity-induced fiber atrophy and slow-to-fast fiber transition, and favors an oxidative phenotype in skeletal muscle of obese rats. Obese Zucker rats were fed gelatin pellets with (15 mg/kg BW/day) or without (placebo group) mangiferin for 8 weeks. Lean Zucker rats received the same gelatin pellets without mangiferin and served as non-obese and non-diabetic controls. Lesser diameter, fiber composition, and histochemical succinic dehydrogenase activity (an oxidative marker) of myosin-based fiber-types were assessed in soleus and tibialis cranialis muscles. A multivariate discriminant analysis encompassing all fiber-type features indicated that obese rats treated with mangiferin displayed skeletal muscle phenotypes significantly different compared with both lean and obese control rats. Mangiferin significantly decreased inflammatory cytokines, preserved skeletal muscle mass, fiber cross-sectional size, and fiber-type composition, and enhanced muscle fiber oxidative capacity. These data demonstrate that mangiferin attenuated adverse skeletal muscle changes in obese rats.

## Introduction

Studies over the past 20 years have described changes in skeletal muscle in human and animal models of both genetic and diet-induced obesity. Muscle atrophy and a switch toward a faster contractile phenotype are well-documented changes in skeletal muscle of obese subjects [1–4]. In addition, an impaired mitochondrial oxidative enzyme capacity has been reported in muscle from obese people and animals [5,6]. However, other studies in patients with Type 2 diabetes and rats with genetic obesity revealed increased mitochondrial density and oxidative enzyme activities in skeletal muscles [7–9]. In addition, studies showed a link between obesity-associated skeletal muscle changes (i.e., decreased muscle mass, slow-to-fast fiber transformation, and impaired mitochondrial function), and the risk of insulin resistance [10–13], suggesting that the prevention of these skeletal muscle changes might improve obesity-induced metabolic dysregulation [14]. For example, it has been widely observed that improving mitochondrial function also improves insulin sensitivity and prevents type 2 diabetes [15].

Chronic inflammation associated with obesity and over-nutrition, characterized by increased levels of inflammatory cytokines such as tumor necrosis factor α (TNF α) and interleukin 6 (IL-6), has been implicated in obesity-induced muscle atrophy via an imbalance in contractile protein synthesis and degradation [3]. Obesity- and diabetes-induced slow-to-fast muscle fiber type transformation has been explained by downregulation of peroxisome proliferator-activated receptors, such as PGC1α and PPARδ [16], which are critical regulators of fiber-type composition [17]. These receptors are typically higher expressed in slow-oxidative type I muscle fibers than in fast-glycolytic type II muscle fibers [18]. It is also known that the activation of the PPARδ-PGC1α axis, together with AMP-activated protein kinase (AMPK), are critical regulators of mitochondrial biogenesis [15,17].

Preliminary work done on mangiferin, a major glucoside of xanthone in the mango plant *Manfigera indica L*., suggests a potential for this pharmacophore as a novel parent compound for treating obesity-related metabolic conditions [19–22]. Evidences show that mangiferin has multiple beneficial biological activities, including potent anti-inflammatory [23–25], anti-oxidant [23,26], and anti-diabetic effects [27–33]. Mechanistic links underlying these actions include inhibition of pro-inflammatory cytokines such as TNF α and IL-6 [23–25], activation of PPARδ and PGC1α receptors [23,34], activation of AMPK [35–38], increments of glucose transporters in adipose and muscle cells [39], and activation of pyruvate dehydrogenase complex [40]. In addition, recent unbiased proteomics studies reveal that mangiferin upregulates proteins pivotal for mitochondrial bioenergetics and downregulates proteins controlling de novo lipogenesis in liver [23,41]. Together, these actions of mangiferin lead to enhancement of carbohydrate utilization in oxidative metabolism, increasing insulin sensitivity and decreasing lipogenesis [22].

A few studies provided evidence that supplementation with mangiferin has beneficial effects on various aspects of muscle function in obese animals. For example, mangiferin supplementation enhanced lipid catabolism in skeletal muscle of these animals by upregulating genes involved in muscle fatty acid β-oxidation [27]. This pharmacophore also induced a shift in muscle respiratory quotient from lipid toward carbohydrate utilization through increased glucose and pyruvate oxidation [40], and by increasing glucose transporters expression and translocation in muscle cells [39]. However, cellular effects of mangiferin on skeletal muscle mass, and contractile and metabolic phenotypes have not been explored in experimental models of obesity.

Based on previous observations supporting mechanistic links of anti-inflammatory, anti-oxidant and anti-diabetic effects of mangiferin, we hypothesized that chronic intake of this pharmacophore should counteract obesity-induced muscle atrophy and slow-to-fast fiber-type switch, as well as to maximize oxidative mitochondrial capacity in skeletal muscle. Thus, the main purpose of this study was to analyze the effect of long-term low-dose mangiferin on size, frequency, and oxidative capacity of skeletal muscle fiber types in obese rats. As an experimental model, we used obese Zucker rats, a well-established model of genetic obesity, insulin resistance, and metabolic syndrome, which were treated with gelatin pellets containing mangiferin (15 mg/kg BW/day) or without mangiferin (placebo) for 8 weeks. Lean Zucker rats served as non-obese and non-diabetic controls. Two muscles with opposite structure and functional significance, the red soleus and the white tibialis cranialis, were examined.

## Results

### Mangiferin improved plasma cholesterol fractions, FGF21 levels, and hyperglycemic response in obese rats

**Fig 1:**
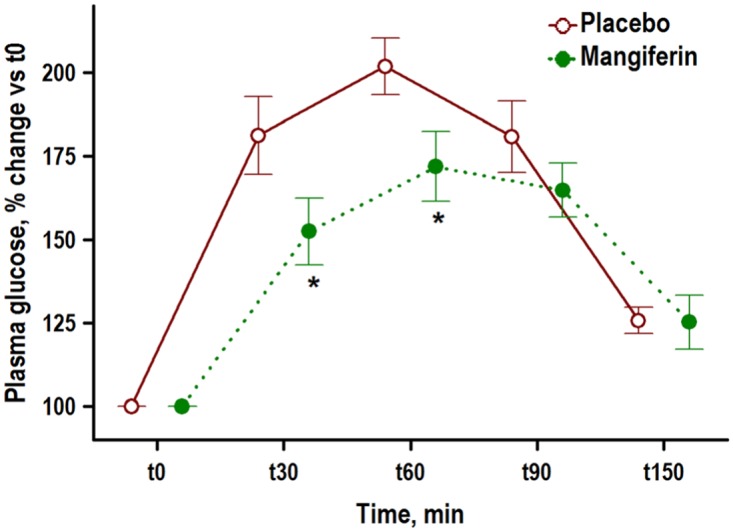
Mangiferin attenuates hyperglycemic response in obese rats after glucose oral administration. Zucker rats with the obese (*fa/fa*) phenotype (2 months old) were treated with placebo or mangiferin (15 mg/kg BW/day) for 8 weeks (n = 10 rats/group), and plasma glucose was monitored every 30 minutes after oral glucose administration (2 g/kg BW). Graph shows means ± SD plasma glucose change vs basal levels over time. * P < 0.05 indicates significant differences between Placebo and Mangiferin groups obtained by unpaired Student’s *t* test.

The effect of mangiferin treatment on plasma metabolites was examined by comparing plasma variables of obese Zucker rats treated with (15 mg/kg BW/day) and without (placebo group) mangiferin, for 8 weeks (Table 1). Mangiferin significantly decreased plasma levels of total cholesterol, LDL cholesterol, and FGF21 levels, and significantly increased serum concentration of HDL cholesterol, but had not effect on serum glucose, triglycerides, insulin, and leptin levels, compared with the placebo group. Despite no effect on final plasma glucose concentration, mangiferin treatment for 8 weeks significantly attenuated hyperglycemic response in a standardized glucose tolerance test compared with the placebo group (Fig 1).

**Table 1:** Mangiferin effects on plasma metabolites in obese rats.

	Lean	Obese Placebo	Obese Mangiferin	P value
Glucose, mg/dl	180 ± 119	183 ± 31	189 ± 21	0.96
Cholesterol, mg/dl	62 ± 7 a	112 ± 8 c	104 ± 10 b	0.00
Chol LDL, mg/dl	4.1 ± 1.4 a	10.2 ± 3.2 c	6.9 ± 3.8 b	<0.001
Chol HDL, mg/dl	25.3 ± 3.2 a	42.3 ± 2.5 b	46.0 ± 3.3 c	0.00
Triglycerides, mg/dl	51 ± 12 a	695 ± 226 b	864 ± 268 b	0.00
Alanine aminotransferase, IU/l	42.6 ± 17.8	54.3 ± 10.2	52.8 ± 8.7	0.09
Adiponectin, μg/ml	2.5 ± 1.3 a	5.1 ± 3.5 b	6.5 ± 1.8 b	0.001
Insulin, ng/ml	0.7 ± 0.4 a	10.6 ± 6.9 b	9.9 ± 4.0 b	<0.001
Leptin, ng/ml	1.1 ± 0.8 a	50.8 ± 9.6 b	60.7 ± 13.1 c	0.00
FGF21, pg/ml	396 ± 230 a	18053 ± 1009 c	9735 ± 1803 b	<0.001

Values are means ± SD, n = 10 rats/group. One–way ANOVA (*P* values denote significance of differences between groups) and Fisher least significant difference post hoc were used to test for differences between pairwise groups; within a row, means with different letters differ significantly (*P*<0.05, at least)

### Mangiferin ameliorated body weight gain, and protected against obesity-induced muscle atrophy

**Fig 2:**
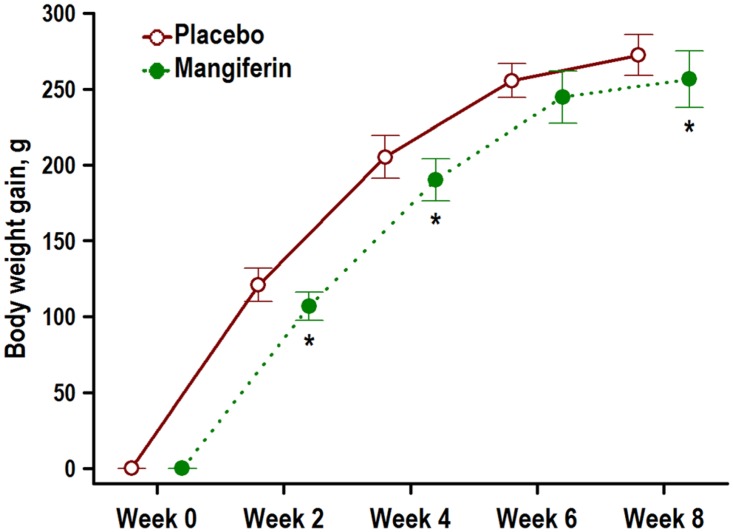
Mangiferin mitigates overweight in obese rats. Zucker rats with the obese (*fa/fa*) phenotype (2 months old) were treated with placebo or with mangiferin (15 mg/kg BW/days) for 8 weeks, and body weight was monitored every two weeks. Graph shows means ± SD body weight gain over time, n = 10 rats/group. * P < 0.05 indicates significant differences between Placebo and Mangiferin groups obtained by unpaired Student’s *t* test.

Daily feed intake was slightly greater in the obese placebo group (mean ± SD, 26.6 ± 1.3 g/d) than in the obese mangiferin group (25.4 ± 1.0 g/d; P = 0.04). Similarly, daily body weight gain was significantly greater in the obese placebo group (4.56 ± 0.24 g/d) than in the obese mangiferin group (4.27 ± 0.33 g/d; P = 0.03). Obese rats treated with mangiferin experienced substantial weight gain reduction compared with the obese placebo group over the 8-week experimental period (Fig 2). Thus, final body weights significantly differed in the three groups (Table 2). Obese rats treated with mangiferin for 8 weeks significantly increased their body weights by 82% compared with lean control rats (P = 0.00), but decreased their body weights by 5% compared with obese control rats (P < 0.01).

**Table 2:** Mangiferin effects on final body weight, muscle weights and Muscle Somatic Index (MSI) in obese rats.

Variable	Lean	Obese Placebo	Obese Mangiferin	P value
Body weight, g	262.9 ± 42.6 a	505.4 ± 14.7 c	479.6 ± 17.6 b	<0.001
M. soleus weigth, mg	156.8 ± 19.0 b	137.5 ± 18.8 a	148.5 ± 14.8 b	<0.01
M. soleus MSI, mg/g	0.60 ± 0.06 c	0.27 ± 0.04 a	0.31 ± 0.04 b	0.00
M. tibialis weight, mg	645.8 ± 86.3 b	550.1 ± 56.3 a	581.8 ± 58.5 a	<0.001
M. tibialis MSI, mg/g	2.48 ± 0.29 b	1.09 ± 0.11 a	1.22 ± 0.14 b	0.00

Values are means ± SD, n = 20 rats/group. One–way ANOVA (*P* values denote significance of differences between groups) and Fisher least significant difference post hoc were used to test for differences between pairwise groups; within a row, means with different letters differ significantly (*P*<0.05, at least)

Despite increased final body weights, wet weights, and muscle somatic indexes (MSI; wet muscle weight referred to body weight) of soleus and tibialis cranialis muscles significantly decreased in obese vs lean control rats (Table 2). M. tibialis wet weight was also significantly lower in the obese mangiferin group than in the lean control group, and it did not differ significantly in mangiferin vs placebo obese rats. However, soleus wet weight and MSIs of both muscles were significantly greater in the obese mangiferin group than in the obese control group.

### Mangiferin preserved muscle fiber cross-sectional size in obese rats

**Fig 3:**
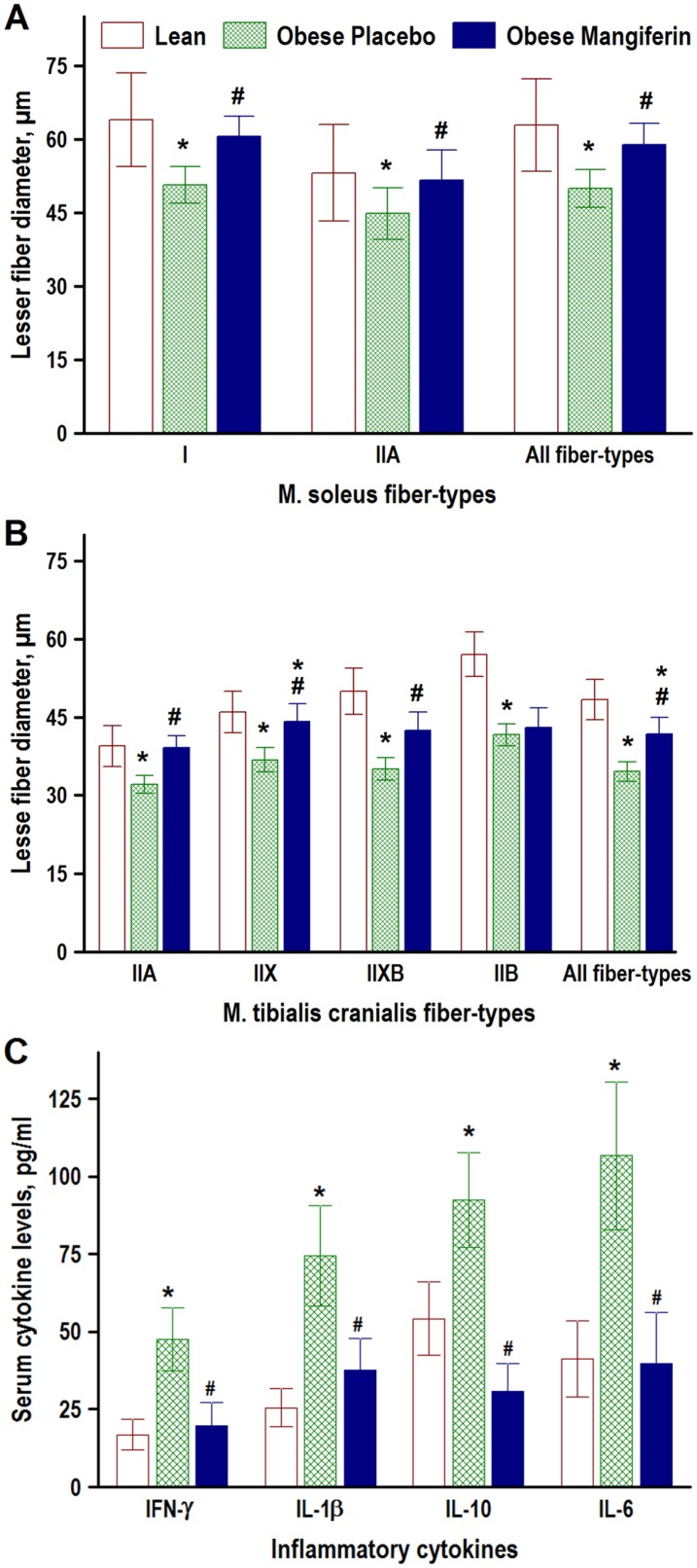
Effect of mangiferin treatment on muscle fiber-size and serum levels of inflammatory cytokines in obese rats. Mean lesser fiber diameters of soleus (A) and tibialis cranialis (B) muscle fiber-types, and plasma levels of interferon-γ (IFN- γ) and interleukin (IL)-1β, IL-6 and IL-10 (C) were determined in lean (*FA*/*Fa* or *FA*/*fa*) Zucker rats and obese (fa/fa) Zucker rats that were treated with either placebo (Obese Placebo) or 15 mg/kg BW/day of Mangiferin for 8 weeks (Obese Mangiferin). For clarity, muscle fiber-types with a percentage below 5% (see Fig 4) are excluded. Values are means ± SD, n = 20 rats/group in A and B, n = 10 rats/group in C. * P < 0.05 vs Lean, # P<0.05 vs Obese Placebo (one-way ANOVA with Fisher LSD post-hoc test).

Mean lesser diameters of main myosin-based muscle fiber types were estimated in soleus and tibialis cranialis muscles, and compared in the three experimental groups Fig 3A and 3B). Soleus muscle fibers were larger than tibialis cranialis muscle fibers. The lower muscle weights observed in the obese control rats (Table 2), corresponded with smaller mean lesser diameters in all soleus and tibialis muscle fiber types (21% and 29% on average, respectively) compared with lean control rats. Mean lesser diameters of soleus and tibialis muscle fiber types were found to be significantly greater (18% and 21%, respectively) in the obese mangiferin group than in the obese control group, but in general (IIB fibers in the tibialis muscle was an exception) were similar (P > 0.05) in obese mangiferin vs lean control rats.

### Mangiferin decreased plasma concentrations of inflammatory cytokines

Because inflammatory cytokines are thought to play a critical role in the genesis of muscle atrophy associated with obesity, we monitored the plasma levels of the inflammatory cytokines interferon (IFN)-γ, interleukin (IL)-1β, IL-6, and IL-10 in the obese rats treated with mangiferin, and compared the results with both obese placebo and lean rats (F. 3C). The serum levels of IFN- γ, IL-1β, IL-6, and IL-10 increased in obese vs lean Zucker rats, but these increments were significantly reversed by treatment with 15 mg/kg BW/day Mangiferin for 8 weeks.

### Mangiferin counteracted obesity-induced switch toward a faster phenotype in white muscle

**Fig 4:**
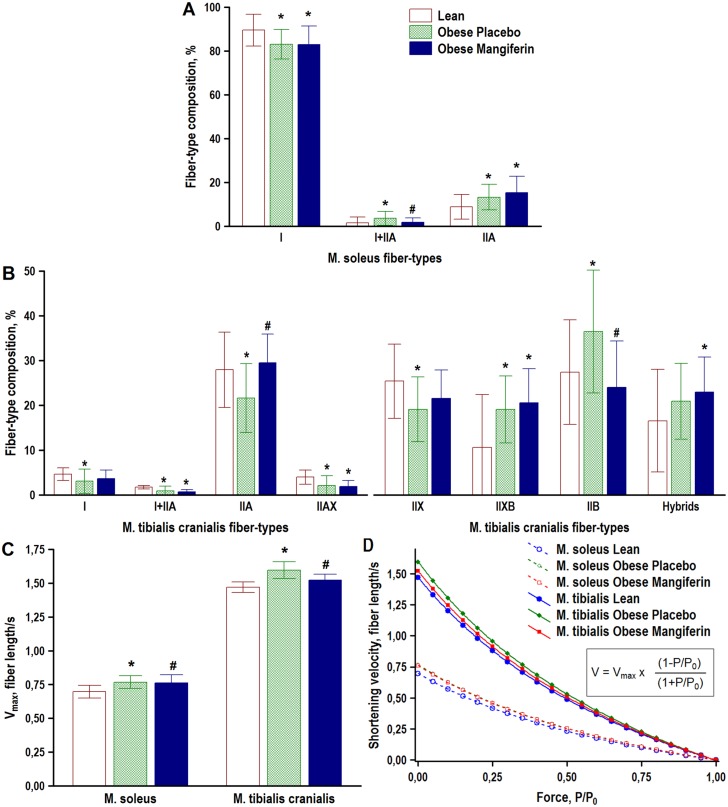
Effect of mangiferin treatment on muscle fiber-type composition, and predicted muscle maximal shortening velocity (V_max_) and the muscle force-velocity relationship in obese rats. The percentages of myosin-based fiber-types (A, B), and predicted V_max_ (C) and force-velocity curves (D), dereived according to the Hill-type mathematical model (*inset*) [42], were determined in Zucker rats with the lean (*Fa*/*Fa* or *Fa*/*fa*) phenotype (Lean), and Zucker rats with the obese (*fa*/*fa*) phenotype that were treated with either placebo (Obese Placebo) or 15 mg/kg BW/day of Mangiferin for 8 weeks (Obese Mangiferin). Bars show means ± SD, n = 20 rats/group. * P < 0.05 vs Lean, # P < 0.05 vs Obese Placebo (one-way ANOVA with Fisher LSD post-hoc test). Data in D are expressed as means of 20 rats/group. V, shortening velocity; _Vmax_, maximal shortening velocity; P, isometric tension; P_0_, maximal isometric tension.

Soleus and tibialis cranialis muscle fibers were typed based on their immunoreactions to antibodies against specific myosin heavy chain isoforms, and the proportions of seven fiber types (I, I+IIA, IIA, IIAX, IIX, IIXB, and IIB) were compared in the three experimental groups (Fig 4A and 4B). All these seven fiber types were identified in the tibialis cranialis muscle, but only three fiber types (I, I+IIA, and IIA) were found in the soleus muscle.

The three experimental groups were not comparable regarding their soleus and tibialis cranialis muscle fiber-type compositions. In the soleus muscle of the two obese groups, slow-twitch type I fiber fractions were reduced by 7%, whereas fast-twitch type IIA fiber percentages were increased by 72% and 49%, respectively, compared with the lean control group (Fig 4A). Type I and IIA fiber percentages in the soleus muscle did not differ in mangiferin vs placebo obese rats. However, in obese rats treated with mangiferin, the percentage of hybrid fibers I+IIA in this muscle was lower than in obese placebo rats (P = 0.04), but comparable to the lean control group (P > 0.05). In the tibialis cranialis muscle of obese placebo rats, there was a significant slow-to-fast fiber-type switching in the direction I → IIA → IIX → IIB, compared with the lean rats (Fig 4B). This was indicated by decreased proportions of type I (34%), I+IIA (47%), IIA (23%), IIAX (50%), and IIX (25%) fibers, and increased frequencies of type IIXB (78%), and IIB (32%) fibers in obese vs lean control rats. The total relative number of hybrid fibers in this muscle was higher in obese than in lean control rats, but this difference did not reach statistical significance (P = 0.16). In general, fiber-type composition of the tibialis cranialis muscle did not differ significantly between the obese mangiferin group and the lean control group, but the percentage of type IIA fibers was higher (37%, P < 0.01), and the proportion of type IIB fibers was lower (34%, P < 0.01) in mangiferin vs placebo obese rats. Total hybrid fibers in the tibialis cranialis muscle were comparable in the two obese groups (P > 0.05), but they were more frequent in the obese mangiferin group than in the lean control group. Together, these data indicated that mangiferin treatment prevented obesity-induced slow-to-fast fiber type transition in the white tibialis cranialis muscle, but not in the red soleus muscle.

The effects of mangiferin on the force-velocity relationship of soleus and tibialis cranialis muscles were estimated according to Hill’s characteristic equation using relative frequencies of myosin-based muscle fiber types in each muscle, and compared in the three experimental groups (Fig 4C and 4D). As expected, predicted maximal shortening velocities (V_max_) were slower in the red soleus muscle than in the white tibialis cranialis muscle. In the soleus muscle, predicted maximal shortening velocities were faster in the obese control group (0.77 ±0.05 fiber length/s) and the obese mangiferin group (0.76 ± 0.06 fiber length/s) than in the lean control group (0.70 ± 0.05 fiber length/s; P < 0.001 in both), but they were comparable in the two obese groups (P > 0.05). In the tibialis cranialis muscle, however, predicted maximal shortening velocities were not comparable in the three groups. V_max_ of this muscle was 9% faster in the obese control group (1.60 ± 0.07 fiber length/s) than in the lean control group (1.47 ± 0.04 fiber length/s; P = 0.00). As shown in Fig 4C and 4D, V_max_ of tibialis cranialis muscle was intermediate in the obese mangiferin group (1.52 ± 0.05 fiber length/s), but significantly different compared with both lean control (P < 0.01) and obese control groups (P < 0.001). Again, these data demonstrate that mangiferin treatment counterbalanced obesity effects on muscle contractile profile, preserving a slower phenotype in white, not red, muscles.

### Mangiferin increased mitochondrial oxidative enzyme activity in obese rats

**Fig 5:**
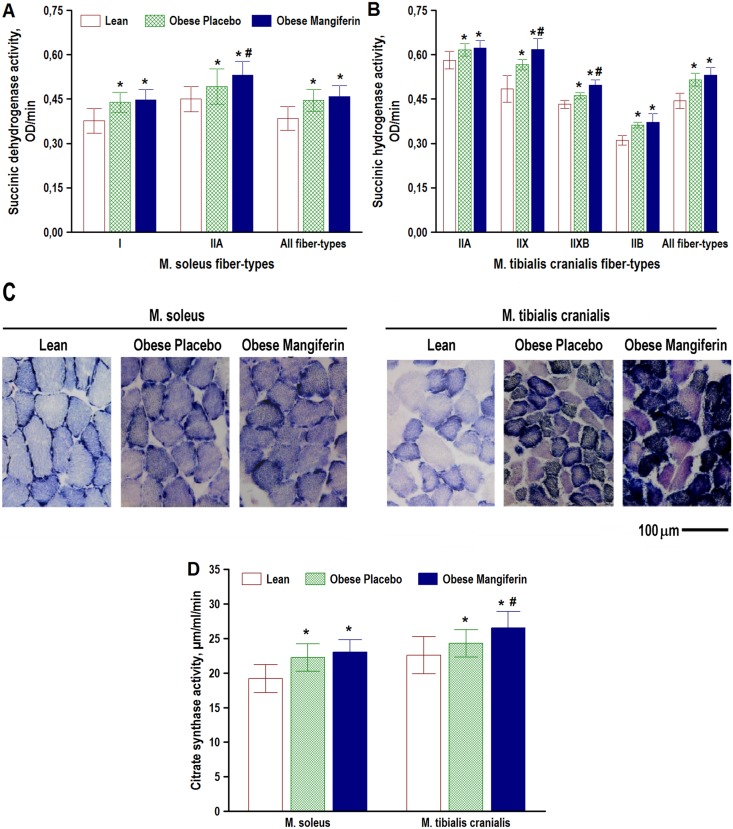
Effect of mangiferin treatment on muscle oxidative capacity in obese rats. The histochemical succinic dehydrogenase activity of individual muscle fiber-types (A, B and C), and the biochemical citrate synthase activity of muscle homogenates (D) were compared in Zucker rats with the lean (*Fa*/*Fa* or *Fa*/*fa*) phenotype (Lean, and Zucker rats with the obese (*fa*/*fa*) phenotype that were treated with either placebo (Obese Placebo) or 15 mg/kg BW/day of mangiferin for 8 weeks (Obese Mangiferin). For clarity, muscle fiber-types with a percentage below 5% (see Fig 4) are excluded in A and B graphs. Values are means ± SD, n = 20 rats/group. * P < 0.05 vs Lean, # P < 0.05 vs Obese Placebo (one-way ANOVA with Fisher LSD post-hoc test). Note in C the darker staining intensity in muscle fibers of both muscles in the two groups of obese rats compared with the lean rat.

To determine the direct effect of mangiferin on muscle oxidative metabolism, we assessed the rate of histochemical SDH enzyme activity within individual muscle fiber types of soleus and tibialis cranialis muscles in the three experimental groups (Fig 5A–5C). The overall mean SDH activity of all muscle fiber types was significantly higher in the two groups of obese rats than in lean animals (soleus, 17%; tibialis, 19%; P < 0.001 in both). This obesity-related improvement of muscle mitochondrial oxidative capacity involved (without exceptions) all soleus and tibialis cranialis muscle fiber types In general, mean SDH activities of muscle fiber types in the two muscles were comparable in mangiferin vs placebo obese groups (P > 0.05). However, specific muscle fiber types, such as type IIA in soleus muscle (Fig 5A), and types IIX and IIXB in tibialis muscle (Fig 5B), exhibited higher mean SDH activities in the obese mangiferin group than in the obese control group (~9%, P < 0.05). This indicates that mangiferin had a direct effect on muscle metabolic profiles, by increasing mitochondrial oxidative capacity of these type II fibers in genetic-obese rats.

The effect of mangiferin treatment on muscle oxidative capacity was also examined by biochemical analysis of the citrate synthase (CS) enzyme activity of soleus and tibialis cranialis muscle homogenates (Fig 5D). In agreement with quantitative histochemical SDH activity in individual muscle fiber-types, whole CS activity of obese placebo rats increased by 16% in the soleus muscle (P < 0.001) and by 8% in the tibialis cranialis muscle (P = 0.03), compared with the lean animals. Moreover, CS activity was comparable in the two groups of obese rats (P > 0.05), but this enzyme activity significantly increased by 9% (P = 0.006) in the obese mangiferin group compared with the obese placebo group.

### Mangiferin had a beneficial overall effect on skeletal muscle phenotypes of obese rats

**Fig 6:**
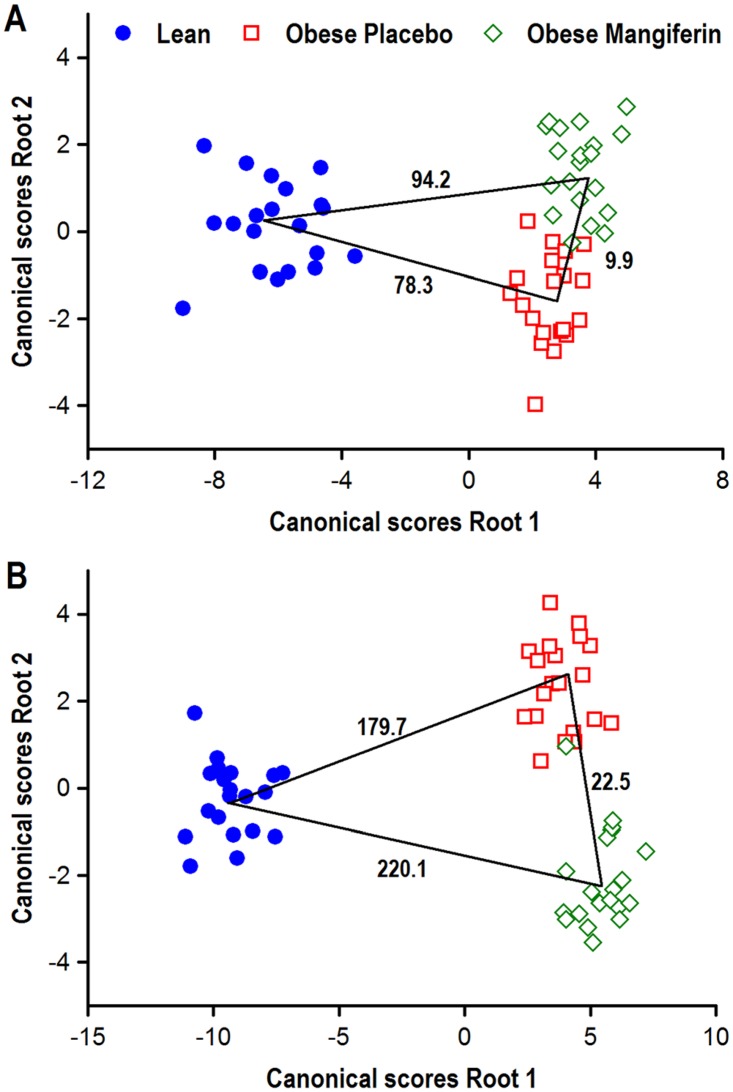
Phenotypic classification of individual hind limb muscles of obese Zucker rats treated with mangiferin in relation to lean and obese controls by means of multivariate discriminant analysis. Individual soleus (A) and tibialis cranialis (B) muscles of all experimental rats were classified into different groups as a function of a cohort of phenotypic muscle characteristics by means of discriminant canonical analysis. The discriminant model for the soleus muscle included the following muscle variables: wet weight, muscle-somatic index, predicted maximal shortening velocity, and percentage, lesser diameter and SDH histochemical activity of type I fibres. The same parameters were included in the statistical model for the tibialis cranialis muscles, but fiber-types IIA, IIX, IIXB and IIB were considered instead of type I fibers. The squares of the Mahalanobis distances between pairs of groups indicative of the overall phenotypic divergences are indicated; P values for the statistical significance of these comparisons between pairs of groups were always significant (P < 0.001).

To summarize quantitatively the degree of similarity or discrepancy of skeletal muscle phenotypes between the three experimental groups, a discriminant canonical analysis was conducted with data available for each muscle (Fig 6). Near all observations (samples) analyzed in soleus and tibialis cranialis muscles (59/60 in both; 98%) were correctly discriminated in their respective experimental groups. Mahalanobis distances between group pairs confirmed significant differences of overall skeletal muscle phenotypes. Ample differences were noted between each group of obese rats (treated or not with mangiferin) and lean rats in the two muscles. Shorter, but significant, distances were also observed between obese rats treated with mangiferin and obese control rats (9.9 in soleus and 22.5 in tibialis; P < 0.001 in both), indicating that mangiferin administration to obese Zucker rats, evoked a significant overall effect on size, contractile, and metabolic features of skeletal muscle fiber types.

## Discussion

The present investigation is to our knowledge the first study addressing the impact of mangiferin administration on skeletal muscle structure and function in obese animals. The main finding was that chronic intake (8 weeks) of low-dose mangiferin (15 mg/kg BW/day) by obese Zucker rats resulted in muscle phenotypes that were significantly different compared with both obese and lean Zucker rats fed a placebo diet. This overall effect included preservation (or reversion) of obesity-induced sarcopenia in red and white muscles, and slow-to-fast fiber-type transition in white, not red, muscles, in addition of increased mitochondrial oxidative capacity in specific type II fibers in both muscle types. Thus, the authors’ initial hypothesis was borne out.

The experiments were designed to administer mangiferin not mixed with the food. This protocol has several advantages: first, the dosing is more exact and daily intake of the dose of mangiferin could be verified; second, and more important, by giving it apart we eliminated any potential effect due to decreased palatability of a mangiferin supplemented diet that could reduce food intake and thus contribute to ameliorate metabolic syndrome. The low dosage of mangiferin used in the present study was effective in obese rats when daily administered over 8 weeks. Beneficial effects of mangiferin were also demonstrated in diabetic rats after intraperitoneal administration of 10 and 20 mg/kg for 14 days [32].

Biochemical results of the present study substantiate and expand previous studies demonstrating effective anti-hyperglycemic (without inducing hypoglycemic state), anti-hyperlipidemic, and anti-atherogenic activities of mangiferin in diabetic animals [32,43]. Recent studies revealed that these beneficial actions of mangiferin are mediated by activation of AMPK signaling pathway, a metabolic sensor that regulates multiple intracellular systems including the cellular uptake of glucose and lipid utilization [35,37,39].

Ample evidence supports that inflammation plays a crucial role in muscle atrophy in patients with obesity and related metabolic dysfunctions [3,12], and that mangiferin decreases circulating markers of inflammation ([23,25], present results). The present study demonstrates for the first time that mangiferin can protect against obesity-induced skeletal muscle mass loss through preservation of myofiber cross-sectional size. Given that mangiferin is a potent anti-inflammatory agent that reduces inflammatory cytokines ([23,25], present results) and obesity-related inflammation promotes muscle atrophy via decreased muscle protein synthesis and increased muscle protein degradation [3], it is not unlikely that the protective effect of mangiferin against obesity-induced muscle sarcopenia could be mediated by inhibition of muscle inflammation signaling.

A central finding of the present study was that mangiferin administration to obese rats resulted in an increased number of type IIA fibers and a decreased number of type IIB fibers in tibialis cranialis muscle when compared to non-supplemented obese rats. This indicates that mangiferin counteracts slow-to-fast fiber transformation in tibialis cranialis muscle of obese rats, which is confirmed by the finding that the predicted maximal shortening velocity of this muscle significantly slowed by ~5% in the obese mangiferin group compared with the obese placebo group. With the sole exception of the hybrid type IIXB fibers, the fiber type distribution of tibialis cranialis muscle was similar between the obese mangiferin and the lean control group. However, the predicted V_max_ of this muscle was significantly faster by ~3.5% in the obese mangiferin than in the lean group, indicating that mangiferin treatment did not counteract all the effects of obesity on muscle contractile profile. Present design is limited to offer mechanistic links underlying variations of muscle fiber types observed in skeletal muscle of obese rats treated with mangiferin. Nevertheless, it is likely that upregulation of peroxisome proliferator-activated receptors (PPARδ and PGC1α) in tibialis cranialis muscle by mangiferin administration could be responsible for the observed difference in muscle fiber types and contractile phenotype of this muscle in obese Zucker rats. This is because mangiferin is a well-known activator of these receptors in diabetic obese rats [34], and downregulation of these receptors is one of the main reasons for the obesity-induced type I-to-type II muscle fiber transition [5,16].

The link between mitochondrial dysfunction and insulin resistance is still a matter of debate, especially in skeletal muscle, the main tissue involved in glucose homeostasis [13]. A key finding of the present study was that the histochemical oxidative activity of individual muscle fibers was increased in all fiber types of red and white muscles in obese versus lean Zucker rats. This histochemical finding was supported by a significant increase in CS activity in sample homogenates of the two muscles (Fig 5D). This finding suggests that insulin resistance can be present without reductions in muscle mitochondrial content, and that obesity provokes insulin resistance increasing muscle oxidative capacity, as previously shown [7].

The present study also revealed that mangiferin improves skeletal muscle oxidative capacity in obese rats through increased SDH activity in specific muscle fiber types. This histochemical result was also supported by the significant increase in CS activity observed in homogenates of the M. tibialis cranialis of obese rats treated with mangiferin compared with obese placebo rats (Fig 5D). An unbiased proteomics study also reported that mangiferin upregulates protein participating in mitochondrial bioenergetics, including the enzyme SDH [41]. As mangiferin is a potent activator of the AMPK-PGC1α pathway [23,34,37,38], and there is ample evidence that PAPRδ-PGC1α receptors are critical regulators of mitochondrial biogenesis (see Ref. [15] for recent review) it is coherent to hypothesize that positive effects of mangiferin on skeletal muscle cell oxidative capacity could be mediated by the activation of this signaling pathway.

In conclusion, our study is the first to demonstrate that mangiferin significantly attenuated adverse skeletal muscle changes in obese rats. This action included preservation of both skeletal muscle mass and muscle fiber-type composition, and enhanced oxidative capacity in muscle fibers. These protective effects of mangiferin on skeletal muscle structure and function were associated with inhibition of inflammatory signaling pathways, but definitive mechanistic links underlying these actions need to be established in further studies. Thus, mangiferin may be a useful dietary component for preventing obesity-associated deleterious skeletal muscle changes, hence improving obesity-induced metabolic dysregulation.

## Materials and methods

### Ethics

All experimental protocols were reviewed and approved by the Ethics Committee for Animal Research of the University of Cordoba (Cordoba, Spain). They followed the guidelines laid down by the Higher Council of Scientific Research of Spain following the normal procedures directing animal welfare (Real Decreto 223/88, BOE of 18 of March) and adhered to the recommendations included in the Guide for Care and Use of Laboratory Animals (US Department of Health and Human Services, NIH) and European laws and regulations on protection of animals, under the advice of specialized personnel.

### Animals and design

Two strains of rats were used in this study: Zucker rats with the obese (*fa/fa*) phenotype (*n* = 40) and Zucker rats with the lean (*Fa/Fa* or *Fa/fa*) phenotype (*n* = 20). All rats were females and aged 8–10 weeks at the beginning of the experiment. Animals (Harlan Laboratories Models, Barcelona, Spain) were individually housed in standard vivarium cages in a temperature–and humidity–controlled environment, with a 12:12–h light–dark cycle and given *ad libitum* access to standard rat diet (Altromin Spezialfutter GmbH, Germany; values per 100 g: energy 351.8 kcal 1100 kJ^–1^, protein content 18%, lysine 1.74%, methionine 1.0%, cysteine 0.31%, tryptophan 0.20%, fat 5%, ash 5.5%, sodium 0.24%, calcium 0.6%, phosphorus 0.6%) and tap water.

Before the experiment began, all rats were maintained for 2 weeks on the standard diet. Afterwards, obese Zucker rats were randomly divided in two groups of 20 rats each. In addition, 20 lean Zucker rats were used for the lean control group. Rats were trained to eat gelatin pellets, which they perceived as a treat. Gelatin pellets were prepared from cooking gelatin (McCormick España SA, Sabadell, Spain) and distilled water using gelatin molds. Each gelatin pellet was made of 160 mg of powdered gelatin and 2 ml water. Two types of pellets which were externally undistinguishable were prepared: pellets containing mangiferin with a purity of 60% and a proved *in vitro* oxygen radical absorbance capacity of 651 μmol TE/g (RG-210, Neuron Bio S.A, Granada, Spain, https://pubchem.ncbi.nlm.nih.gov/compound/Mangiferin#section=Top) and pellets without mangiferin (placebo). The amount of mangiferin within each pellet was adjusted for each rat to provide a dose of 15 mg/kg BW. Rats in the lean control and the obese placebo groups were maintained for 8 weeks on the standard diet and received the placebo pellets daily. Obese rats in the treatment group received the standard diet and the pellets with mangiferin daily for 8 weeks. Rats were fed ad libitum and the pellets (mangiferin or placebo) were administered once daily in the morning. Food intake was recorded daily and the animals were weighed every two weeks.

### Blood biochemistry

One week before the end of the experiment (week 7) glucose tolerance tests were performed in a subset of rats (n = 10 rats/group) in the two groups of obese rats. Briefly, rats were fasted, a baseline blood sample was obtained and an oral dose (2 g/kg) of glucose (Acofarma, Tarrasa, Spain) was administered in the form of gelatin pellets. Pellets were prepared as above described and were supplemented with glucose instead of mangiferin. Again, the amount of glucose added to the gelatin pellet was adjusted individually to provide a dose of 2g/kg. Subsequently 4 blood samples were drawn at 30, 60, 90 and 150 minutes after eating the glucose pellet. Blood samples were obtained by puncture of a tail vein and glucose was measured in whole blood using a glucometer (Arkray Factory Inc, Japan).

At 8 weeks, all rats were sacrificed by aortic puncture and exsanguination under deep general anaesthesia (sodium thiopental 50 mg kg^–1^, Penthotal^®^, Abbot, Illinois, USA; ip). Blood samples were obtained from the abdominal aorta in heparinized syringes at the time of the euthanasia. Plasma separated by centrifugation was used for measurements of glucose, cholesterol and triglyceride levels by spectrophotometry (Biosystems, Barcelona, Spain), insulin and leptin concentrations by radioimmunoassay (Millipore, St. Charles, MO, USA), and fibroblastic growth factor 21 (FGF21) by ELISA (Millipore, St. Charles, MO, USA).

### Determination of plasma cytokine levels

The plasma samples were thawed on ice, gently vortexed and then centrifuged at 13,200 rpm for 10 min at 4°C immediately prior to testing. The released cytokines (IL-1β, IL-6, IL-10 and IFNγ) were measured by using the Bio-Plex Pro^™^ Rat Standard Kit (Bio-Rad Laboratories), following the manufacturer’s instructions, and the Bio-Plex 200 system. Data were analyzed using Bio-Plex Manager software, version 6.0 (Bio-Rad), and a five-parameter logistic curve fit.

### Muscle sampling and tissue preparation

Muscle samples were obtained at the time of sacrifice. Soleus and tibialis cranialis muscles were dissected and individual muscles were wet weighted. These muscles were selected as two representative muscles of a typical red, slow–twitch muscle (soleus, composed primarily of slow–twitch muscle fibers) and a characteristic white, fast–twitch muscle (tibialis cranialis, composed primarily of fast–twitch muscle fibers in its white region), respectively [44]. Also because these two hind limb muscles are opposite regarding their resting functional activities, and have a different energetic dependence on blood borne substrates (see Ref. [44]).

Upon collection, tissue blocks from the muscle belly were mounted on cork blocks with the use of OCT embedding medium (Tissue–Tek II, Miles Laboratories, Naperville, IL, USA) and oriented so that myofibers could be cut transversely. Specimens were systematically frozen by immersion in isopentane (30 s), kept at the freezing point in liquid nitrogen, and stored at –80°C until analyzed. Muscle samples were routinely frozen between 2 and 4 min after removal, because it has been demonstrated that the interval between removal and freezing has a significant (negative) effect on skeletal muscle fiber size [45]. All muscle sampling and muscle preparation procedures were always carried out by the same investigator, experienced in skeletal muscle biopsy studies, taking care to standardize both the location and the freezing of the sample.

### Myosin Heavy Chain (MHC) immunohistochemistry

Muscle samples were serially sectioned (10–μm–thick) in a cryostat (Frigocut, Reitchert Jung, Nubloch, Germany) at –20°C and used for immunohistochemistry. Immunohistochemistry was performed with five monoclonal antibodies specific against MHC isoforms: BA–D5 (DMS, Braunschweig, Germany; anti–MHC–I), SC–71 (DMS; anti–MHC–IIa), BF–35 (DMS; anti–MHCs–I plus–IIa and–IIb), S5–8H2 (Biocytex Biotechnology, Marseille, France; anti–MHCs–I plus–IIx and–IIb), and BF–F3 (DMS; anti–MHC–IIb). The specificity of these monoclonal antibodies for MHCs in rat skeletal muscle has previously been reported [46–48]. The immunoperoxidase staining protocol with avidin–biotin complex (ABC) protocol was used as previously described [49].

### Quantitative enzyme histochemistry

Additional serial sections were used for quantitative enzyme histochemistry. The activity of the enzyme succinate dehydrogenase (SDH, EC 1.3.5.1), used as an oxidative marker, was determined on 10–μm–thick sections, by using quantitative histochemical methods previously adjusted and validated in rat skeletal muscle [49].

### Image analysis and morphometry

Sections were examined in a blind fashion by the same investigator (L.M.A.), who had experience of the normal appearance of mammalian skeletal muscle fibers. All serial sections for immunohistochemistry and enzyme histochemistry were visualized and digitized as previously described [50]. A region containing between 450 and 650 fibers was selected for further analyses. In the tibialis cranialis muscle, this area was selected from the core of the white (superficial) portion of the muscle, since it contains a higher number of fast–twitch muscle fibers (98%) than the red (deep) portion (93%) of the muscle [44]. Images were saved as digitized frames at 256 gray levels. The gray levels were converted to optical density (OD) units by using a calibrated set of OD filters. The digitized images of the fibers in the histochemical reaction (SDH) within the selected region were traced manually and analyzed for the lesser fiber diameter and the average OD for each individual muscle fiber. The average fiber OD for each histochemical reaction was determined as the average OD for all pixels within the traced fiber from three sections incubated with substrate minus the average OD for all pixels of the same fiber from other two sections incubated without substrate [49]. Because a number of factors can influence the reliability of histochemical enzyme activity determinations, we checked the variability on three consecutive sections for the SDH histochemical reaction by repeated measurements of the same individual fibers. Only coefficients of variation for triplicate measurements of ODs below 5% were accepted in the present study; this demonstrated the high analytical precision that can be achieved for the measurement of fiber OD on enzyme histochemical sections.

The fibers in the selected area were classified according to their MHC content by means of visual examination of immunostainings of the five serial sections stained with the battery of anti–MHC monoclonal antibodies as previously described [46,47]. The reactivity of each individual muscle fiber in these five consecutive sections was judged as positive or negative by comparing the intensity of the reaction of neighbouring fibers. Seven fiber types were categorized, four of them as pure fibers expressing a unique MHC isoform (i.e., type I, IIA, IIX and IIB), and other three as hybrid phenotypes co–expressing two different MHC isoforms (type I+IIA, IIAX and IIXB).

The relative frequency of different muscle fiber types in the selected region was used to numerically express the fiber type composition of each muscle sample. The lesser fiber diameter of the same fibers was averaged according to fiber type. Individual SDH ODs of muscle fibers were averaged according to the MHC muscle fiber–type and used for statistical analyses. For minor fiber types (I+IIA and IIA in the soleus muscle, and I, I+IIA and IIAX in the tibialis cranialis muscle), there were so few fibres in most muscle samples that a statistically reliable determination of their lesser diameter and SDH activities was impossible. In consequence, muscle fibre–types showing, on average, a fibre percentage below 5% were excluded from these analyses. A polymorphism index for obese and lean animals was calculated in each muscle by dividing the number of hybrid fibers expressing more than one MHC isoform by the total number of fibers in the muscle sample [51].

### Prediction of muscle shortening velocity

In an attempt to provide a functional assessment of the variation of myosin-based muscle fiber distribution on muscle’s shortening speed, we used the following equation to predict the maximal shortening velocity (V_max_ Muscle) of soleus and tibialis cranialis muscles [51], according to the mathematical model proposed by A. V. Hill [52]:

$$V\text{max}Muscle=\sum_{}^{}\frac{\%ft\times V\text{max}ft}{100}$$

Where %_*ft*_ *i*s the proportion of each fiber type in *Muscle*, and *V*_*max ft*_ is the maximal shortening velocity for a given fiber type (fiber length s^-1^). Values from Botinelli et al. [53] were used for *V*_*max ft*_, and *V*_*max ft*_ of hybrid fibers were averaged between *V*_*max*_ of their respective pure fiber-types. In this model, soleus and extensor digitorum longus muscles were used as representative slow-twitch and fast-twitch muscles, respectively [42]. Fiber-type composition are comparable in rat extensor digitorum longus and tibialis cranialis muscles [44].

### Whole muscle Cintrate Synthase (CS) activity

The activity of the enzyme citrate synthase (CS, EC 4.1.3.7), a mitochondrial enzyme and marker of muscle oxidative potential, was measured from whole homogenates by using fluorometric technique described elsewhere [54]. The activity of this enzyme was calculated in units of μmol NADH converted per minute per gram of freeze-dried muscle.

### Statistical analyses

All statistics and charts were run on Statistica 7.0 for Windows (StatSoft I, Statistica, Data software System, www.statsoft.com). Muscle sample was the unit of analysis for the present dataset (S1 dataset). A total of 120 muscle samples (60 animals x two muscles–soleus and tibialis cranialis) were available for statistical analysis. Sample size and the power of a contrast of hypothesis were estimated by power analysis and interval estimation of the statistical software employed. Accepting an α –risk of 0.05 and a β –risk of 0.2 in a two–sided test, 20 subjects/group were considered necessary to recognize as statistically significant a difference greater than or equal to 0.05 OD units for SDH histochemical activity between any pair of groups assuming that 3 groups exists. The common standard deviation (SD) was assumed to be 0.06 and it was anticipated a drop–out rate of 0%. Normality of muscle variables was tested using a Kolgomorov–Smirnov test and data were expressed as means ± SD. Unpaired t-test were used to test for differences of plasma variables between groups and before and after treatment with either mangiferin or placebo. One-way ANOVA was used to test for differences of muscle variables between groups at the end of the experimental period. When either a significant (P < 0.05) or a marginal (0.05 < P < 0.1) effect was observed, the Fisher least significant difference post hoc test was used to locate specific significant differences between pairwise groups.

Overall differences among experimental groups were estimated by squared Mahalanobis coefficients provided by multivariate discriminant analyses of the two hind limb muscles. This distance take into account all muscle fiber-type variables summarizing the overall phenotype of each individual muscle sample, allowing its classification into one of the three groups. These coefficients served to compare overall muscle characteristics of group pairs to establish their homologies and differences regarding the control skeletal muscle phenotype.

## Supporting information

S1 Dataset Full dataset included in the study. Include all data used and analyzed in the present study.
(XLSX)
